# Prevalence and Characterization of Breakthrough Pain Associated with Chronic Low Back Pain in the South of Spain: A Cross-Sectional, Multicenter, Observational Study

**DOI:** 10.1155/2018/4325271

**Published:** 2018-04-23

**Authors:** Luis Miguel Torres, Antonio Javier Jiménez, Ana Cabezón, Manuel Jesús Rodríguez

**Affiliations:** ^1^Pain Unit, University Hospital Puerta del Mar, Cádiz, Spain; ^2^Kyowa Kirin Farmacéutica SLU, Madrid, Spain; ^3^Pain Unit, Regional University Hospital of Málaga, Málaga, Spain

## Abstract

Chronic low back pain (CLBP) is highly prevalent in industrialized countries, where it is one of the main causes of disability. Patients with CLBP in treatment with opioids often experience episodes of breakthrough pain (BTP), but data on prevalence and treatment preferences are scarce. The objectives of this study were, first, the evaluation of the prevalence of BTP in patients with CLBP in the South of Spain (*N* = 1,868) and, second, the characterization of BTP in these patients (*N* = 295). Data was collected on presence of BTP, type and location of pain, treatment, compliance, and patient satisfaction. We found a prevalence of BTP in patients with CLBP of 37.5% (95% CI: 35.3%–39.7%), similar in men and women. 75% of the patients were older than 50 years. The preferred drug of patients who control BTP with opioids is fentanyl (78.3%) and its most common form of administration is nasal (53.2%). Therapeutic compliance was high and 46.3% of patients considered the control of their BTP very satisfactory. Our study showed that BTP is common in patients with CLBP and that current treatments seem adequate.

## 1. Introduction

Chronic low back pain (CLBP) is defined as localized pain between the lower limit of the ribs and the lower limit of the buttocks that lasts for more than 12 weeks. In CLBP the intensity of pain varies depending on postures and physical activity and is usually accompanied by severe limitation of the movement [1, 2]. However, CLBP is frequently associated with pain crises characterized by high intensity and intermediate duration, also known as breakthrough pain (BTP). BTP is a transient exacerbation of pain that occurs spontaneously, in relation to either a specific predictable or unpredictable trigger, despite relatively stable and adequately controlled pain [3]. BTP episodes generate an increase in pain that lasts about half an hour to an hour and which can seriously interfere with the patients' quality of life as well as their functional capacity [4, 5].

The prevalence of BTP in cancer patients has been widely described and ranges from 33% to 89% [5, 6]. In Spain the prevalence, characteristics, implications, and modalities of treatment of BTP have been described for chronic oncologic pain in Catalonia, showing that episodes of BTP occurred in 41% of these patients [7]. However, BTP in nononcologic and chronic diseases has been poorly studied and is still questioned [8]. Studies of non-cancer-related BTP in different populations have shown prevalence varying between 48% and 74% [5, 6, 9, 10]. A more recent study of patients treated with opioids in the United States showed that up to 80% of these patients had regular bouts of BTP [11]. For these patients BTP associated with negative outcomes: patients with BTP had more pain-related interference in function, worse physical and mental health, more disability, and worse mood. Among patients presenting BTP, the most common syndrome is low back pain (52%) [5]. Clearly, this and other studies clearly suggest that BTP could be highly prevalent in all populations of patients treated with opioids, deserves wider recognition, and needs special treatment plans.

CLBP prevalence data ranging from 13% to 50% have been reported [12], and in Spain it has been estimated that there was prevalence of CLBP of up to 24.2% in women and 12.3% in men aged 65 years or older [13]. However, the prevalence and characteristics of BTP among CLBP patients are unknown. It is possible that some physicians underrecognize the occurrence of BTP in patients with persistent pain [4]. For this reason, this regional study was carried out with the objective of determining the prevalence of BTP among patients with CLBP visiting the Pain Units of large hospitals in the Autonomous Communities of Andalucia and Melilla in the South of Spain (2016 population = 8.402 million). Additionally, this study sought to obtain other relevant data, such as the prevalence of BTP in relation to the origin of pain, type of pain, and therapeutic approaches for its control. The evaluation of this information is of interest to better understand this pathology and improve the management of pain in these patients.

## 2. Methods

This is an observational, multicenter, cross-sectional epidemiological study of BTP in patients with CLBP. The study was conducted in the Pain Units of 20 hospitals in the South of Spain (Autonomous Communities of Andalucia and Melilla) between July and December of 2015. The main objective was to assess the prevalence of BTP in patients with CLBP and the secondary objectives were to characterize BTP based on etiology, pathology, and other clinical characteristics (type of BTP, number of daily episodes, duration and intensity, and management of pain) and to evaluate the prevalence of each of the different causes of pain.

### 2.1. Patient Selection

The selection criteria for the study of prevalence were (a) age of patients >18 years; (b) patients with chronic pain secondary to opioid-controlled CLBP; (c) patients with adequate oral and written comprehension; and (d) informed consent of the patient.

In this study, cross-sectional data collection was carried out for 3 months in each of 20 participating centers, all large hospitals throughout the Autonomous Region of Andalucia and Melilla. The population of this area of Spain was 8.388 million in 2016. The hospitals participating in the study were the Carlos Haya Hospital (324 patients, 17.3% of the total); Poniente H. (301, 16.1%); Virgen del Rocío H. (180, 9.6%); Ciudad de Jaén H. (160, 8.6%); Comarcal Melilla H. (149, 8.0%); Virgen de la Victoria H. (143, 7.7%); Puerto Real University H. (123, 6.6%); SAS de Jerez H. (122, 6.5%); Guadix H. (101, 5.4%); Reina Sofía H. (78, 4.2%); Nuestra Señora de Valme H. (75, 4.0%); Baza H. (63, 3.4%); Puerta del Mar H. (42, 2.2%); Torrecárdenas H. (5, 0.3%); and Virgen Macarena University H. (2, 0.1%). 1,868 patients were surveyed in the study of BTP prevalence.

The prevalence study included all the data recorded by the researchers in the prevalence sheets during the established period. In the study of the secondary objectives, all the patients that met the selection criteria established in the protocol (295 patients) were included. Twelve patients were excluded from the analysis of the characterization of BTP because they did not meet the selection criteria.

Informed consent was obtained from all individual participants included in the study. All procedures performed were conducted in accordance with the 1964 Declaration of Helsinki and its later amendments or comparable ethical standards. The study was approved by the Ethics Coordinating Committee for Biomedical Research of Andalucia on May 4, 2015 (study code: ADD-DOL-2015-1).

### 2.2. Methodology and Data Collection

In the prevalence study the following variables were collected: presence of BTP (yes/no), sex, and age. The prevalence of BTP was calculated as the number of patients with BTP over the total number of patients surveyed. Secondary variables collected included types of BTP (incidental, spontaneous), number of BTP episodes in the last month, origin, cause, location, average time until BTP relief, average duration of episodes and baseline, and rescue treatments.

Each investigator identified, over a period of 3 months, patients with CLBP on treatment with opioids who came to the Pain Unit. The patients were specifically asked about the presence of BTP. Patients included in the study were the first two patients of each day who met the inclusion criteria and gave their informed consent. During a single visit the researcher collected the data and variables of the study. Data collection was performed for 3 months in each participating center. Given their observational nature, the data were obtained from the patient's medical record and/or directly from the patient and according to the physician's usual clinical practice. Pain intensity of patients with BTP was assessed using a visual analogue scale (VAS). Data collection was done using Data Collection Notebooks on paper. The data was stored in a relational database on a MySQL server. The database was protected by an SSL security certificate for the correct encryption of the data. The database was endowed with security margins and internal consistency rules to avoid the entry of incorrect data or of anomalous or inconsistent values.

### 2.3. Statistical Methods

All patients who fulfilled the selection criteria and gave their informed consent were included in the statistical analysis of the study. The prevalence of BTP with its respective 95% confidence interval was calculated as the percentage of subjects who presented BTP among the number of patients with opioid-controlled CLBP collected by each investigator in the frequency sheet of patients over 3 months. This prevalence was also adjusted by age and sex group. Given the descriptive nature of the study, the statistical methodology used was based mainly on an exploratory analysis of the data by means of the calculation of descriptive parameters. Categorical variables were presented as absolute frequencies and relative frequencies. The possible association between the intensity of BTP according to the VAS scale and the type of BTP (incidental or spontaneous) was analyzed by the Student *t*-test.

## 3. Results

This cross-sectional observational study analyzed the prevalence of BTP in patients with CLBP who visited the Pain Unit of large hospitals in Andalucia and Melilla (Spain). A total of 1,868 patients were included, of whom 25% were under 50 years of age, 50% were between 50 and 71 years old, and 25% were older than 71 years. 36.1% of the patients included in the prevalence study were men and 63.9% were women. In the study of BTP characterization 295 patients were included and the mean age was 61.5 years (45.1% men and 54.9% women).

We determined that the prevalence of BTP in patients with CLBP is 37.5% (95% CI: 35.3% −39.7%) in this population. The prevalence in men and women was similar, 39.6% and 36.4%, respectively. There were no statistically significant differences between the presence or absence of BTP as a function of sex (*p* value = 0.178, Fisher's exact test) and age (*p*-value = 0.95, Student's *t*-test).

Regarding CLBP characteristics, 74.2% of the patients had chronic pain of mixed origin, 7.5% neuropathic, and 18.3% nociceptive. Radiating pain was reported by 53.2% of the patients and referred pain by 20.7% of the patients. Regarding the characteristics of BTP, 61.4% of patients described it as being of mixed type, whose main location was low back (39.0%) or low back and lower limbs (34.2%) (Table 1). The average degree of BTP was 84.4 points as measured by the visual analogue scale (VAS).

With regard to the frequency and duration of the BTP, 59.1% of the patients surveyed had 10 or more episodes of BTP in the month prior to data collection. The duration of seizures was less than 45 minutes in 78.7% of patients; time to pain relief was less than 15 minutes in 66.5% of cases, and 21.8% of patients suffered more than 5 daily crises (Table 2).

To treat chronic baseline pain (CLBP), 100.0% of patients used opioids and 50.5% were also medicated with nonsteroidal anti-inflammatory drugs (Table 3). The most widely used opioids were tapentadol (28.1%), oxycodone (17.3%), oxycodone/naloxone (17.3%), tramadol (15.9%), and fentanyl (13.9%). Other drugs used to treat chronic basal pain were anticonvulsants (62.9%), dual antidepressants (20.3%), and muscle relaxants (13.5%). 56.6% of patients used other treatments against chronic pain, such as local anesthetic peripheral injections (51.5%), local anesthetic nerve injections (38.9%), TENS (8.4%), and physiotherapy (6.0%) (Table 3).

With regard to the treatment of BTP, 81.4% of the patients used opioids, mainly fentanyl (78.3%) and tramadol (12.9%) (Table 4). The preferred route of administration of fentanyl was nasal (53.2%) (Table 5).

Compliance for both CLBP and BTP was always high and above 90%. In the case of CLBP the medication was taken “generally” or “always” by 98.3% of the patients; in the case of treatment of BTP the “generally” or “always” responses were given by 91.2% of the patients (Table 6). The most frequent causes for patients not taking the medication for chronic pain were forgetfulness (38.0%) and side effects (34.0%), while noncompliance by patients presenting BTP were mainly due to side effects of medication (35.5%) and forgetfulness (20.4%) (Table 6).

Regarding the evaluation of the degree of satisfaction with the treatment, the “very satisfactory” response was superior to 40% for both the treatment of basic pain and that of BTP, being slightly higher in the latter (46.3%). However, the response was “somewhat unsatisfactory” or “very unsatisfactory” in 12.4% of the patients for the treatment of CLBP and of 17.3% for the treatment of BTP (Table 7). Our results show that both compliance and patient satisfaction are generally high, suggesting that the quality of assistance in the treatment of BTP seems adequate in this population.

## 4. Discussion

The main objective of this epidemiological study was to determine the prevalence of breakthrough pain (BTP) associated with chronic low back pain (CLBP) in patients who visited the Pain Units of hospitals in Andalucia and Melilla, in the South of Spain. We have determined that the prevalence of this type of pain is 37.5% (95% CI: 35.3%–39.7%). In addition, we have characterized these types of pain and their treatment in the region. This study has allowed us to know and quantify the degree of compliance and satisfaction of patients with regard to pain treatments, thus enabling future therapeutic interventions and better management of the problem.

Opioids are useful drugs that can be used in the treatment of CLBP, although careful dosage and monitoring of adverse effects such as constipation, nausea, pruritus, dizziness, drowsiness, and tolerance should be monitored [2]. Normally, doses remain stable at low levels for years with the objective of minimizing a possible increasing tolerance caused by chronic use. Not only is the treatment of CLBP aimed at an adequate control of pain, but also the relief is translated into a reduction of the limitation of the daily activity that it generates. In our study, compliance with the treatment of chronic pain was around 98%. When medication was not taken, the most frequent causes were forgetfulness (38.0%) and side effects (34.0%).

Despite their usefulness, the benefits and risks of opioids for patients with chronic pain have been a matter of debate, and specially their administration in the management of BTP. An adequate assessment of BTP should include frequency and duration of episodes, intensity and type of pain, precipitating factors, prior medication, and the effectiveness of rescue therapy. Clues about patterns of BTP in specific patients can be obtained from the patient record and even better from a “pain diary” in which the patient records the episode immediately [14]. In our opinion, an adequate management of BTP should be based on three aspects: prevention, anticipation, and use of appropriate medication. Strategies for treatment of BTP can be nonpharmacological (such as educational measures to promote habits that reduce the risk of BTP episodes) or pharmacological (analgesic treatment) [15, 16]. Compliance with the treatment of BTP was as high (90.0%) in the case of chronic pain, but in this case the main reason for not taking the medication was side effects (35.0%). The objective of the control of BTP, together with the knowledge of its evolution and its rapid treatment, is to avoid its negative effects on the functional and psychological state of patients, as well as improve their quality of life [16]. The majority of patients in our study treated BTP with opioids (81.4%) and, of these, 78.3% did so with fentanyl. The preferred route of administration was nasal (53.2%). A recent comparative review of the routes of administration of fentanyl showed that fentanyl administered nasally generates more rapid analgesia than oral or transmucosal administration for cancer patients [17]. In these patients, both oral and nasal transmucosal administration of fentanyl have been shown to be an effective treatment because of its potent analgesic effect, rapid action, and sustained effect [18]. Oral fentanyl can be effective and safe treatment for BTP but, as it is the case with all chronic opioid treatments, appropriate patient selection, administration, dosing, and monitoring must be applied. [19–22].

The limitations of the study are those derived from the design of the study, as it is a cross-sectional analysis where the frequency of patients with BTP associated with CLBP who were referred to the Pain Units may not be representative of the general population. This would occur if only certain patients and not all those with CLBP and/or BTP come to these units. Also, some of the parameters analyzed are subject to the effects of patient self-report, lack of coded diagnosis, and the evaluation of comorbidities. Additionally, the study was geographically restricted to certain centers of the Autonomous Communities of Andalucia and Melilla, and extrapolation of the results to the national population could be problematic.

In this study we estimated the prevalence of BTP in the population visiting the Pain Units of hospitals in Andalucia and Melilla. Epidemiological data on BTP has allowed us to know the preferred treatments and the level of satisfaction of the patients. These parameters will undoubtedly help in the assessment of pain management in this group of patients and the possible improvement of future therapeutic interventions.

## Figures and Tables

**Table 1:** Characteristics of BTP in patients with opioid-treated CLBP.

	*N*	%^*∗*^
Type of BTP		
Neuropathic	49	16.6
Nociceptive	60	20.3
Mixed	181	61.4
NA	5	1.7
Neuropathic^1^		
Incident	27	55.1
Idiopathic	19	38.8
NA	3	6.1
Nociceptive^2^		
Incident	52	86.7
Idiopathic	8	13.3
Pain localization		
Low back	115	39.0
Low back + lower limbs	101	34.2
Lower limbs	29	9.8
Low back + gluteal/hip	11	3.7
Low back + dorsal	6	2.0
Gluteal/hip	4	1.4
Low back + upper limbs	2	0.7
Other	10	3.4
NA	17	5.8

^*∗*^Percentage calculated over the total of patients (*N* = 295). ^1^Percentage calculated over the total number of patients with neuropathic BTP (*n* = 49). ^2^Percentage calculated over the total number of patients with nociceptive BTP (*n* = 60); NA = not available.

**Table 2:** Characteristics of BTP episodes.

	*N*	%
Number of BTP episodes last month^1^		
1–5	46	15.7
5–10	74	25.3
10–15	65	22.2
>15	108	36.9
Duration of BTP episodes (minutes)^1^		
1–14	56	19.1
15–29	80	27.3
30–45	95	32.4
>45	62	21.2
Time until pain relief (minutes)^2^		
1–5	30	10.3
6–10	99	33.9
11–15	65	22.3
16–30	49	16.8
>30	49	16.8
Number of episodes per day^1^		
0	23	7.8
1–5	206	70.3
≥5	64	21.8

^1^Percentage calculated over the number of patients with information available (*N* = 293);  ^2^percentage calculated over the number of patients with information available (*N* = 292).

**Table 3:** Treatments for basal pain (CLBP).

	*N*	%
NSAIDs	149	50.5^1^
Opioids^1^	295	100.0^1^
Tapentadol	83	28.1
Oxycodone	51	17.3
Oxycodone/naloxone	51	17.3
Tramadol	47	15.9
Fentanyl	41	13.9
Buprenorphine	17	5.8
Morphine	14	4.7
Codeine	2	0.7
Hydromorphone	1	0.3
Other pharmacological treatments^2^	251	85.1^1^
Anticonvulsants	158	62.9
Dual antidepressants	51	20.3
Muscle relaxants	34	13.5
Tricyclic antidepressants	19	7.6
Neuroleptics	17	6.8
Corticoids	12	4.8
Bisphosphonates	10	4.0
Calcitonin	1	0.4
Spasmodic	1	0.4
Others	62	24.7
Nonsystemic pharmacological and nonpharmacological treatments^3^	167	56.6^1^
Acupuncture	3	1.8
Local anesthetic nerve injections	65	38.9
TENS	14	8.4
Physiotherapy	10	6.0
Local anesthetic peripheral injections	86	51.5
Spinal stimulation	3	1.8
Other	25	15.0

*Note*. patients could receive more than one treatment. ^1^Percentages calculated over the total number of patients (*n* = 295). ^2^Percentages calculated over the total of patients with pharmacological treatments for basal pain (*n* = 251). ^3^Percentage calculated over the total of patients with nonpharmacological treatments for basal pain. NSAIDs = nonsteroidal anti-inflammatory drugs; TENS = transcutaneous electrical nerve stimulation.

**Table 4:** Treatments for BTP.

	*N*	%
Opioids^1^	240	81.4
Morphine	13	5.4^2^
Fentanyl	188	78.3
Oxycodone	17	7.1
Tramadol	31	12.9
Other opioids	2	0.8
Oxycodone/naloxone	1	50.0
Tapentadol	1	50.0
Other pharmacological therapies^1^	60	20.3
Metamizole	26	43.3^3^
Paracetamol	13	21.7
Ibuprofen	6	10.0
Dexketoprofen	5	8.3
Metamizole (Nolotil)	4	6.6
Lidocaine	2	3.3
Other	11	18.7

*Note*. Patients can receive more than one treatment. ^1^Percentages calculated over the total number of patients (*N* = 295). ^2^Percentages calculated over the total number of patients treated with opioids for BTP (*N* = 240). ^3^Percentages calculated over the total number of patients treated with other drugs for BTP (*N* = 60).

**Table 5:** Route of administration of opioids for BTP.

	*N*	%
Morphine		
Oral, short-acting	11	84.6
Nasal	1	7.7
Oral, long-acting	1	7.7
Fentanyl		
Nasal	99	53.2
Sublingual	52	28
Transmucosal	23	12.4
Oral, short-acting	9	4.8
Intrathecal	2	1.1
Transdermal	1	0.5
Oxycodone		
Oral, short-acting	13	81.3
Oral, long-acting	3	18.8
Tramadol		
Oral, short-acting	26	86.7
Oral, long-acting	4	13.3

**Table 6:** Evaluation of adherence to treatment.

	CLBP		BTP	
	*N*	%	*N*	%
Investigator				
Global adherence to medication				
Always	211	72.8	165	58.3
Generally	71	24.5	90	31.8
Sometimes	6	2.1	19	6.7
Never	1	0.3	8	2.8
NA	1	0.3	1	0.4
Patient				
Do you take your medication as prescribed?				
Always	240	82.8	189	66.8
Generally	45	15.5	69	24.4
Sometimes	5	1.7	15	5.3
Never	0	0.0	9	3.2
NA	-	-	1	0.4
Reason for not taking medication				
Forgot	19	38.0	19	20.4
Adverse effects	17	34.0	33	35.5
Difficulty of administration	2	4.0	5	5.4
Other	1	2.0	12	12.9
NA	11	22.0	24	25.8
Do you take medication not prescribed by your physician?				
No	234	80.7	253	89.4
NA	23	7.9	9	3.2
Yes	33	11.4	21	7.4
Paracetamol	8	24.2	4	19.0
Metamizole	4	12.1	7	33.3
NSAIDs	4	12.1	-	-
Ibuprofen	3	9.1	-	-
Other	12	36.0	7	33.6

NA = not available; NSAIDs = nonsteroidal anti-inflammatory drugs.

**Table 7:** Evaluation of satisfaction with treatment.

	CLBP		BTP	
	*N*	%	*N*	%
Very satisfactory	120	41.2	131	46.3
Somewhat satisfactory	135	46.4	103	36.4
Somewhat unsatisfactory	32	11.0	32	11.3
Very unsatisfactory	4	1.4	17	6.0

## References

[1] Balagué F., Mannion A. F., Pellisé F., Cedraschi C. (2012). Non-specific low back pain. *The Lancet*.

[2] Delitto A., George S. Z., Van Dillen L. (2012). Low Back Pain. *Journal of Orthopaedic & Sports Physical Therapy*.

[3] Bennett D. S., Simon S., Brennan M., Shoemaker S. A. (2007). Prevalence and characteristics of breakthrough pain in patients receiving opioids for chronic back pain in pain specialty clinics. *Journal of Opioid Management*.

[4] Payne R. (2007). Recognition and diagnosis of breakthrough pain. *Pain Medicine*.

[5] Portenoy R. K., Bennett D. S., Rauck R. (2006). Prevalence and Characteristics of Breakthrough Pain in Opioid-Treated Patients With Chronic Noncancer Pain. *The Journal of Pain*.

[6] Gatti A., Mediati R. D., Reale C. (2012). Breakthrough pain in patients referred to pain clinics: The Italian pain network retrospective study. *Advances in Therapy*.

[7] Gómez-Batiste X., Madrid F., Moreno F. (2002). Breakthrough cancer pain: Prevalence and characteristics in patients in Catalonia, Spain. *Journal of Pain and Symptom Management*.

[8] Manchikanti L., Singh V., Caraway D. L., Benyamin R. M. (2011). Breakthrough pain in chronic non-cancer pain: Fact, fiction, or abuse. *Pain Physician*.

[9] Portenoy R. K., Bruns D., Shoemaker B., Shoemaker S. A. (2010). Breakthrough pain in community-dwelling patients with cancer pain and noncancer pain, Part 2: Impact on function, mood, and quality of life. *Journal of Opioid Management*.

[10] Zeppetella G., O'doherty C. A., Collins S. (2001). Prevalence and characteristics of breakthrough pain in patients with non-malignant terminal disease admitted to a hospice. *Palliative Medicine*.

[11] Narayana A., Katz N., Shillington A. C. (2015). National Breakthrough Pain Study: Prevalence, characteristics, and associations with health outcomes. *PAIN*.

[12] Wong A. Y., Karppinen J., Samartzis D. (2017). Low back pain in older adults: risk factors, management options and future directions. *Scoliosis and Spinal Disorders*.

[13] Jiménez-Sánchez S., Fernández-de-las-Peñas C., Carrasco-Garrido P. (2012). Prevalence of chronic head, neck and low back pain and associated factors in women residing in the Autonomous Region of Madrid (Spain). *Gaceta Sanitaria*.

[14] McCarberg B. H. (2007). The treatment of breakthrough pain. *Pain Medicine*.

[15] Webster L. R. (2008). Breakthrough pain in the management of chronic persistent pain syndromes. *The American Journal of Managed Care*.

[16] Taylor D. R., Webster L. R., Chun S. Y. (2007). Impact of breakthrough pain on quality of life in patients with chronic, noncancer pain: Patient perceptions and effect of treatment with oral transmucosal fentanyl citrate (OTFC®, ACTIQ®). *Pain Medicine*.

[17] Davis M. P. (2011). Fentanyl for breakthrough pain: A systematic review. *Expert Review of Neurotherapeutics*.

[18] Rogríguez D., Urrutia G., Escobar Y., Moya J., Murillo M. (2015). Efficacy and Safety of Oral or Nasal Fentanyl for Treatment of Breakthrough Pain in Cancer Patients: A Systematic Review. *Journal of Pain and Palliative Care Pharmacotherapy*.

[19] Fine P. G., Narayana A., Passik S. D. (2010). Treatment of Breakthrough Pain with Fentanyl Buccal Tablet in Opioid-Tolerant Patients with Chronic Pain: Appropriate Patient Selection and Management. *Pain Medicine*.

[20] Busse J. W., Craigie S., Juurlink D. N. (2017). Guideline for opioid therapy and chronic noncancer pain. *Canadian Medical Association Journal*.

[21] Chou R., Fanciullo G. J., Fine P. G. (2017). Clinical guidelines for the use of chronic opioid therapy in chronic noncancer pain. *The Journal of Pain*.

[22] Manchikanti L., Kaye A. M., Knezevic N. N. (2017). Responsible, Safe, and Effective Prescription of Opioids for Chronic Non-Cancer Pain: American Society of Interventional Pain Physicians (ASIPP) Guidelines. *Pain Physician*.

